# The COVID-19 pandemic in children and young people during 2020-2021: A complex discussion on vaccination

**DOI:** 10.7189/jogh.11.01011

**Published:** 2021-12-25

**Authors:** Igor Rudan, Davies Adeloye, Vittal Katikireddi, Josie Murray, Colin Simpson, Syed Ahmar Shah, Chris Robertson, Aziz Sheikh, EAVE II collaboration

**Affiliations:** 1 Centre for Global Health, Usher Institute, The University of Edinburgh, Edinburgh, UK; 2 MRC/CSO Social & Public Health Sciences Unit, Glasgow, UK; 3 COVID-19 Surveillance Lead, Public Health Scotland, Fife, UK; 4 School of Health, Wellington Faculty of Health, Victoria University of Wellington, New Zealand; 5 Usher Institute, The University of Edinburgh, Edinburgh, UK; 6 Department of Mathematics and Statistics, University of Strathclyde, Glasgow, UK and Public Health Scotland, Glasgow, UK

Vaccination of children and young people against COVID-19 remains highly debated, with considerable policy divergence internationally. Vaccinating younger age groups was not an initial plan when the original variant of the SARS-CoV-2 virus emerged, because children and young people (CYP) seemed very mildly affected by COVID-19. However, new mutations led to increased virulence of the SARS-CoV-2. This led to an increase in the population threshold of vaccination coverage required for prevention of viral spread, possibly to levels above 80% vaccine uptake in the whole population. Also, due to the successful roll-out of vaccination to older and at-risk populations, the virus began to circulate in greater numbers amongst younger populations, which became a new concern. An important element was also the issue of harms unrelated to health, such as educational disruption. A broader perspective weighted the long-term educational benefits and shorter-term health issues. In addition, increasing awareness of the possible rare complications of COVID-19 in CYP and the “long COVID” syndrome prompted the scientific study and comparison of the risks of disease vs the safety of vaccination, even among the very young [1,2].

As a result, pharmaceutical and biotechnological companies started testing the efficacy and safety of vaccines in adolescents and children. In parallel, an increasing number of countries started to roll out vaccines in under-18-year-olds to protect themselves from the new and ever-more-virulent variants. The first studies on the uptake, effectiveness and safety of the vaccines in children at the population level were conducted in 2021 [1-4]. This editorial aims to review the development of research on vaccinating CYP: the circumstances, rationale and evidence that was used to extend the vaccine programme to CYP during the COVID-19 pandemic in some countries. The paper is based on the information available until December 1, 2021.

## ETHICAL AND LEGAL ASPECTS OF VACCINATING MINORS

Considerations about possibly vaccinating particularly vulnerable children have increased interest in ethical and legal aspects of vaccinating minors and ensuring consent appropriately [3-17]. It emerged from previous literature that, to enrol children in any clinical trials, some knowledge gaps would need to be addressed. A clear rationale was needed for vaccines in different groups of children. Then, the effects of immune maturation should be well understood from birth to the age of 18 years [7]. Ethical concerns would need to be carefully documented and addressed. Notably, specific situations and needs of children with developmental disorders and chronic conditions should be analysed, health inequities considered [8], and vaccine hesitancy assessed in each society and community before attempting vaccination programmes [7].

Given that COVID-19 is typically a mild disease among minors, a risk-benefit ratio required to enrol children in vaccine trials and any other clinical trials should be carefully considered. There are real differences in innate and adaptive immunity between children and adolescents, which may affect vaccine efficacy [7]. Early ethical considerations have concluded, based on appropriate frameworks, that there are strong ethical reasons to vaccinate the young to protect the old, as well as those children, should not be excluded and allowed to get infected, and that this argument should also be considered in policy-making [4,11]. While forming vaccination policies and designing programs, it was expected for the risks imposed on children to be reasonable and carefully considered [4,5,13]. However, there were also opposing views expressed on the ground of ethics and not everyone agreed [10,12]. Interestingly, the debate over ethics extended to participants of human challenge trials, and arguments were even made that “healthy young adults” may not be the best first choice for such trials in COVID-19, although they are considered to have the lowest risk, but that they should be replaced with “healthy older adults” (ie, those over 30 years of age) [9].

The discrepancy in the views arose because many questions about children were initially unanswered, such as their role in the transmission, the effectiveness of the vaccine in reducing transmission, and the expected benefits to any vaccinated child. When deciding on approving and mandating a novel vaccine for a disease that is not a major threat to children, all relevant underlying scientific evidence on disease severity, vaccine safety and vaccine effectiveness must be very strong. Alternatives and the levels of coercion associated with each should also be carefully considered. When planning vaccination programmes, difficult issues of balancing between self-interest and duty to others also come into consideration. Requirements outlined for mandatory vaccination against COVID-19 in children, from an ethical perspective, should include information on whether the disease is a sufficient threat, positive comparative expected usefulness of vaccination, and proportionate coercion [6]. The case for the mandatory vaccine in children may also be quite strong for influenza vaccination during the COVID-19 pandemic [6].

There was also interest in whether vaccination against COVID-19 in children should ever become mandatory. The literature about school mandates is abundant, while it is more limited for adults. However, there is a less principled body of common understanding of whether is it appropriate to mandate a specific vaccine. An ethical framework to consider when should any school mandates apply suggests that a principled and rather neutral way to think of a vaccine mandate is to consider it as an intersection of competing values while examining the factors that strengthen or weaken each of the values under the circumstances. The framework should take into account the values of autonomy, beneficence, utilitarianism, justice, and non-maleficence because they could all be affected by mandating immunization [3,14-16].

When considering pediatric off-label use of COVID-19 vaccines from an ethical and legal point, some authors contextualized ethical and legal precedents regarding off-label use and offered an analysis of the ethical permissibility of, and considerations for, pediatric off-label Covid-19 vaccination based on individual benefits, risks, and available alternatives. Their analysis challenged the ethics of a blanket prohibition on off-label pediatric COVID-19 vaccination. The argument is that it limits clinicians’ ability to provide care that they determine is clinically and ethically appropriate. Inevitably, COVID-19 “creates population-level ethical considerations that are at times in tension with individual health interests” [17].

## CONSIDERATION OF INCLUSION OF CHILDREN AND YOUNG PEOPLE IN VACCINE TRIALS AND VACCINATION PROGRAMMES AGAINST COVID-19

Lack of clearly effective treatment, the emergence of Multisystem Inflammatory Syndrome in Children (MIS-C), the presence of particularly vulnerable children and young people in the population, the many unknowns of the burden of “long COVID-19”, and the improved understanding of ethical and legal aspects of vaccinating minors against COVID-19 have all led to an intense, vigorous and still ongoing debate in the scientific literature on whether minors should also be mass-vaccinated in the COVID-19 pandemic [18-102]. Parents were called upon by experts to do their “due diligence” and continue to inform themselves in making the best decision for their children [19]. Some were quite clear in pointing that delaying vaccine clinical trials in children will also delay global recovery from COVID-19 and that the consequence will be an unnecessary prolongation of negative impacts on children's education, health, and emotional well-being, while also denting hopes for equitable access to opportunities for development and social success [21,25].

Tested vaccine platforms were analysed and practical considerations for their potential use in children were reviewed, along with the ethical aspects [22]. Modelling was done to assess the potential impact of vaccinating children on the course of the pandemic [43,90]. Concerns were also expressed about the negative effects of the pandemic on routine childhood vaccine coverage, especially in many places of the world where they are most needed to protect the most underprivileged children from vaccine-preventable diseases [22]. This was not only relevant to low- and middle-income countries, but also applied to some areas of the high-income world, such as Texas, USA, where a 47%-58% decline in immunization rates was reported between 2019 and 2020 in 5-16 months old children [44].

Reasons for and against vaccinating minors were carefully weighted in many of the scientific papers on this issue. European Centre for Disease Prevention and Control (ECDC) issued a report on June 1, 2021, which contained important considerations for public health authorities in the EU and European Economic Area, focused on vaccinating children and adolescents. It suggested that the decisions need to be made after considering the uptake in older age groups, the incidence of COVID-19 in the general population, and practical issues concerning availability and access to vaccines globally. The ECDC report stated that minors “would experience few direct benefits from being vaccinated; rather, the goal would be to increase overall population immunity and reduce transmission” [48,102].

Nevertheless, the concrete evidence on the exact role of this age group in the transmission has been rather patchy and not always pointing to the same conclusion, depending on the population and the wave that was studied, and the anti-epidemic measures that were in place. And even within those who were <18 years old, there seemed to be an age gradient, with younger children seemingly less susceptible to infection and less likely to pass it on, suggesting vaccination of older age groups first [48]. In terms of priorities for protecting, children with cancer, disorders of the heart, diabetes, hypertension, neurological conditions, or kidney disease, with a risk of hospitalisation, would be prioritised – but again, the evidence was rare because they were already being shielded very well [48].

Besides those apparent arguments of reducing the overall transmission to protect the elderly who are most vulnerable, and protecting the children who are most at-risk themselves, other arguments included the uncertain long-term sequelae of COVID-19 and multisystem inflammatory syndrome (MIS-C). Proponents of mass-vaccination of children suggested that life with less concern over contracting or passing the infection would improve children’s and adolescents’ mental health and well-being, allowing them to resume education and social interactions important for their development. Also, it would prevent this age group from becoming a “pocket” of the population in which SARS-CoV-2 would continue to circulate freely and potentially mutate into more dangerous variants. However, at which exact point should children in high-income countries be prioritised for vaccination over vulnerable adults in LMICs remained a matter for serious ethical and practical debate [48].

In parallel to this debate, vaccine trials in different age groups of minors began by the leading COVID-19 vaccine developers. This led to authorisations of COVID-19 Pfizer-BioNTech’s mRNA BNT162b2 vaccines for the use among the adolescents aged 16-18 years, followed by 12-15 age group, and finally, 5-11 years old children in Canada, the USA, and then Europe. On May 5, 2021, Canada was the first country to approve the COVID-19 vaccine for emergency use in children aged 12-15 years; later the same month, the FDA and European Medicines Agency also gave their approvals to the Pfizer-BioNTech COVID-19 vaccine for adolescents. Approvals for 5-11-year-olds by the FDA had followed on October 29, 2021. Several days earlier, an agency advisory committee reviewed data from a clinical trial that tested a low-dose version of the Pfizer–BioNTech vaccine on this age group and voted almost unanimously for recommending its emergency approval [48,51].

These authorisations were based on a clear efficaciousness, immunogenicity and safety in children in those age groups [48,51] Double-blind, randomised, controlled, phase 1/2 clinical trials showed that the inactivated COVID-19 vaccine (CoronaVac) had good safety, tolerability, and immunogenicity in youths aged 3-17 years, inspiring the trials of other COVID-19 vaccines in minors of very early age. BNT162b2 vaccine has shown efficacy as high as 100% in children aged 12-15 years [51]. The CDC concluded that the benefits of vaccination outweigh the risks for COVID-19 vaccination in adolescents and young adults [98].

In a useful joint statement of the European Academy of Paediatrics and the European Confederation for Primary Care Paediatricians [84], the position was given on various aspects of vaccine implementation among the minors. Their conclusion was a strong message that “action was necessary to clarify the future of SARS-CoV-2 vaccination in children and adolescents at different levels”. This came after the American Academy of Pediatrics, the Advisory Committee on Immunization Practices and the Canadian Pediatric Society already advocated for the vaccination of all children and adolescents older than 12 years [92,99,100], while the United Kingdom held back this decision because there could simply find no sufficiently clear evidence about the safety and need of vaccination against SARS-CoV-2 in children [101].

The European Center for Disease Prevention and Control also took a position that, due to the limited benefits in children and adolescents, “careful consideration of the epidemiological situation and vaccine uptake in older age groups should be given before targeting children and adolescents” [102]. The European Pediatric Societies over Severe Acute Respiratory Syndrome Coronavirus 2 (SARS-CoV-2) Vaccination in Children warned that a trial of AstraZeneca's COVID-19 vaccine in children aged 6-17 years was started in March 2021 in the UK, but it had to be paused as a precautionary measure because of reports on blood clots in adults. Therefore, European Pediatric Societies recommended against giving the vaccine to children younger than 12 years before “rigorous clinical trials are completed, adverse events carefully assessed, and not until vaccines are authorized and adequate dosage established by the respective national agencies”, as this would ensure that vaccines were safe and effective for this age group [72]. With similar caution, vaccination was endorsed in principle as a positive and safe public health measure by the Expert Panel on Infectious Diseases and COVID-19 of the Global Pediatric Pulmonology Alliance (GPPA), as well as the number of relevant Chinese medical and pediatric authorities [79,83].

By July 2021, several countries have already made decisions to move ahead with vaccinating their children to curb the epidemic wave that was affecting them at the time. Most notable examples were Israel, Canada, the USA, United Arab Emirates, Chile, Cuba and China. In Malta, close-knit family structures and adolescents having frequent contacts with their grandparents and travelling abroad for a school affected the decision. However, as mentioned earlier, vaccine advisers in the United Kingdom have recommended that only particularly vulnerable adolescents, or those living with vulnerable adults, should be vaccinated at that stage of the pandemic [57].

Some countries were quick to vaccinate children to try to avoid the further need for school closures, which was always a really difficult decision. Relative to their risk of contracting the disease, children and adolescents have been disproportionately affected by lockdown measures. Within household outbreaks, children didn’t seem responsible for the majority of household introductions of the virus. The impact of the reopening of schools on community transmission remained a matter of scientific debate. However, it was still difficult to justify why some countries prioritized the opening of retail and hospitality sectors while leaving their schools closed. Also, the cost of lower skills attainment would harm nations in the years to come, while protecting children from COVID-19 by closing schools has led to an increase of their hospitalizations due to accidents at home in some countries [2].

Because of uncertainty over the scientific evidence required to answer several important questions, it is clear that many professional societies preferred a cautious approach to introducing mass vaccination among those younger than 18 years. Others argued that there was simply no time to wait for the perfect evidence and that it won’t matter if it comes after most children have already been infected. One way of reasoning was to clearly explain that COVID-19 is a childhood illness, just as well as an illness of the adults. Only in the USA, millions of children have been infected with SARS-CoV-2 and at the end of October 2021, it was estimated that about 100 000 children were being infected each week in the USA. As a result, tens of thousands of children have been hospitalized, and about one-third did not have any pre-existing medical conditions. Eventually, almost 700 children have died from COVID-19 in the USA, while not a single child was reported to have died from vaccination by that time. Pfizer’s mRNA vaccine was 90.7% effective against symptomatic COVID-19. Vaccine-associated myocarditis has been a mild and self-limited side-effect, which is quite different from the cardiovascular problems that can be caused in children by COVID-19 or MIS-C [94]. So, essentially, it comes down to a question – between the two risks, which one should the carers choose? Although the risk posed by COVID-19 in children is very low, the vaccine still seems to be a much lesser risk, even without taking into account its other benefits [94].

## CAREGIVERS’ WILLINGNESS TO VACCINATE MINORS, OPINIONS AMONG ADOLESCENTS AND VACCINE HESITANCE

The work on ethical and legal aspects and the debate on the costs and benefits of vaccinating minors have led to another important area of research: using representative population-based surveys to establish caregivers’ willingness to vaccinate minors and their vaccine hesitance [103-134]. In November 2020, one of the first such surveys was performed by the International COVID-19 Parental Attitude Study (COVIPAS) Group. They surveyed 1541 caregivers who brought their children to 16 pediatric emergency departments across six countries during the first pandemic wave, from March 26 to May 31, 2020. At that point, 65% of caregivers intended to vaccinate their child against COVID-19 once a vaccine is available. The most common reason was to protect their child (62%), while among the hesitant ones the most common reason was the vaccine's novelty (52%) [103].

This early investigation was then followed by many further surveys that used similar methodology and explored this issue in different global settings. It emerged that 18% of parents would enrol their child in a clinical trial for a COVID-19 vaccine, while 14% would agree to a randomized placebo-controlled study. Those parents were also more willing to be enrolled in a COVID-19 vaccine trial themselves, have an older child, have children who received all vaccinations according to schedule in their country, and have high school education or lower. Mothers were less likely to enrol their child in a trial than fathers [104].

In the US, in March 2021, 49% of parents who had a child ≤12 years of age had plans to vaccinate them when this becomes possible. Those with lower income and less education were more hesitant, quoting safety concerns and lack of need [118]. In New York City, in March and April 2021, 62% of parents had plans to vaccinate their children under the age of 12 years, 15% did not have such plans and 23% were unsure [121]. These results were similar across the US because further representative studies also reported 46%-49% of parents willing to vaccinate their children, with vaccine side effects (62%) and vaccine safety (49%) being the most significant concerns affecting hesitancy [115,117,123]. The parents were more likely to vaccinate their children if they were older children if parents had a bachelor's degree or higher education, if they received or were likely to receive the vaccine themselves, or if they voted for Democrats. The most trusted source of information was the child's doctor [123].

In China, parents' willingness to vaccinate their children was high from the start, at 70%-87% [105,120,122,133]. However, among 941 children with special diseases, only 50% of parents were willing to provide vaccination for their children [134]. The perception that a family member would support them in having their children take up COVID-19 vaccination was an important factor in their decision [105]. Caregivers who were male, aged 40-49 and from the rural area were more willing to vaccinate their children, while a history of adverse vaccine reactions and allergies in children led to hesitancy [120]. More than 80% of the caregivers expressed a high level of trust in vaccine information released by official and health-related agencies [120]. Several studies in China conducted at a later date in the pandemic found slightly different results [114,124], where only 45%-53% would likely or very likely to have their children aged <18 years vaccinated. The issues were perceived vaccine efficacy, perceived protection duration, perceived likelihood that China would prevent another wave of COVID-19 outbreak with vaccines, and willingness to receive a COVID-19 vaccination themselves. In China, it seemed that information exposure through social media was associated with higher parental acceptability of COVID-19 vaccination while knowing anyone who had serious side effects following COVID-19 vaccination would deter them from vaccinating their children [114,124].

The KUNO-Kids health study in Europe found that 58% of parents intended to get vaccinated against COVID-19 themselves, while 51% intended to have their child vaccinated. Those willing typically also had stronger confidence in their knowledge on prevention measures and had beliefs that policy measures against COVID-19 were not exaggerated [110]. In Italy, 60% of the caregivers were inclined to vaccinate, while 30% were still considering this option and 10% were hesitant. Hesitancy rates were the highest in female caregivers under 30 years of age who had children aged 6-10 years, lower educational level, who relied on information found on the internet and social media, who dislike mandatory vaccination [108].

In a study in South Korea, 76.5% of parents intended to get vaccinated and 64.2% intended to have their children vaccinated, but only 49.6% of children responded that they would get COVID-19 vaccination [125]. Among European adolescents, a history of allergies, migration background and female gender were associated with greater vaccine hesitancy [126,127]. In Qatar, the parents of 12-year-old adolescents were more hesitant (22%) compared to the 13-year-olds (16%) and 15-year-olds (15%). Moreover, parents of adolescents from Gulf countries (97% Qatari) were more hesitant (35%) as compared to the other nationalities. Also, parental vaccine hesitancy rates in Qatar were higher when adolescents suffered from chronic disease, or who were previously COVID-19 infected [128].

When asking US-based adolescents about their preferences, 65%-76% reported willingness to receive a COVID-19 vaccine [106,111], whereby about 40% were unconditionally prepared and about 30% were conditional, if experts thought that vaccination was safe and helpful. They would trust the opinions of medical organizations such as CDC and the WHO (42%) and health care professionals (32%), while they were concerned about the side effects (36%) and efficacy (20%). There were ethnic differences, with Asian American adolescents being more, and African Americans less willing to get vaccinated than the rest [106]. Willingness also differed by physical health conditions, COVID-19 knowledge, practising physical distancing and adversity history [111]. In Chinese adolescents [119], the same proportion - 76% - would accept COVID-19 vaccination. Those more likely to agree were younger adolescents who have heard about COVID-19 vaccines, thought that they could protect them from COVID-19 infection, and believed that vaccines are safe. An interesting experiment showed that conveying strong social norms to young people leads to reduced hesitancy and stronger intentions, but norms did not lead to different effects in comparison to standard vaccine information from the authorities. Moreover, young people were not more strongly influenced by norms when the reference group were other young people, rather than the society in general [113].

In England, 27 910 students from 180 schools were asked about vaccine hesitancy between May and July 2021 and 50% would take a vaccination, 37% were undecided, while 13% would opt-out. Younger students were considerably more hesitant, with 38% of 9-year-olds, 51% of 13-year-olds and 78% of 17-year-olds choosing to opt-in. Those hesitant would more likely belong to deprived socioeconomic contexts with higher rates of home rental vs homeownership, they were more likely to smoke or vape, use social media more, and feel that they did not belong in their school community more often. The study suggested that efforts to increase vaccination uptake may be necessary for these age groups [130]. Another study found that 42% of 9^th^ graders reported they are willing to get a COVID-19 vaccine, while 22% were “a little hesitant”, 21% were “somewhat hesitant”, and 15% were “very hesitant” [131]. In a Canadian survey, 63% of parents intended to vaccinate their children against COVID-19. Those more hesitant were employed part-time, their children did not receive influenza vaccine, and they were more likely to believe that COVID-19 vaccination was unnecessary [132].

Among paediatricians and family physicians, changes were noted to routine childhood vaccination delivery as a result of the COVID-19 pandemic. In October-December 2020, for children aged 0-2 years, 5% of paediatricians and 15% of family physicians reported they had stopped vaccinating these children at any time. For children aged 4-6 years, 19% of paediatricians and 17% of family physicians reported stopping routine vaccinations at any time, and for children aged 11-18 years, the proportion was 24% and 19%, respectively. By the year 2021, nearly all of them returned to pre-pandemic vaccination services [107].

Some authors showed that there is a difference in endorsement of vaccines depending on the use of subjective or objective language. About 53% of guardians in the US-based study endorsed vaccine administration for their children early in the pandemic when subjective language was used, but this proportion decreased to 46% later in the pandemic; however, when objective language was added, acceptance increased to 69%. The most common reasons for hesitance were concerns about adverse effects and safety [129].

A study in Canada identified social inequalities in COVID-19 vaccine acceptance and uptake for children and adolescents, suggesting that special efforts will be required to reach underprivileged populations. Children from households with annual incomes <US$100 000 had an 18% lower prevalence of being vaccinated, or being very likely to get vaccinated, compared to household incomes ≥US$150 000. Moreover, vaccine-eligible adolescents from the most deprived neighbourhood were half as likely to be vaccinated compared to those from the least deprived neighbourhood [109].

## VACCINE SAFETY AND EFFICACY TRIALS IN MINORS

Based on previous research, the importance of including children and young people in COVID-19 therapeutic trials was highlighted by numerous authors [135-168]. Their arguments were that waiting too long to enrol minors could deny them and their families the benefits of a vaccine without a proper justification. It could also delay an effective response to the pandemic unnecessarily. However, it was also recognised that enrolling minors were also potentially associated with yet unknown risks. Soon after safety and immunogenicity were established in adults, vaccine trials in children and adolescents have also started [138].

One of the first published trials of vaccines in young people was on the safety and immunogenicity of the SARS-CoV-2 BNT162b1 mRNA vaccine in younger and older Chinese adults. It was a randomized, placebo-controlled, double-blind phase 1 study in a single-centre in Jiangsu province, China [141]. It was a parallel-group, double-blind phase 1 trial of the vaccine candidate BNT162b1 in 144 healthy SARS-CoV-2-naive Chinese participants. The study concluded that BNT162b1 has an acceptable safety profile and produces high levels of humoral and T cell responses in an Asian population [141]. By the summer of 2021, the Chinese group of authors published a study on the safety, tolerability, and immunogenicity of an inactivated SARS-CoV-2 vaccine (CoronaVac) in healthy children and adolescents aged 3-17 years. It was a double-blind, randomised, controlled, phase 1/2 clinical trial at Hebei Provincial Center for Disease Control and Prevention in Zanhuang. The vaccine was given by intramuscular injection in two doses, spaced for 4 weeks. The primary safety endpoint was adverse reactions within 4 weeks after each shot, while the primary immunogenicity endpoint was the seroconversion rate of neutralising antibody to live SARS-CoV-2 at 4 weeks after the second shot. Between Oct 31 and Dec 2, 2020, the authors enrolled 72 participants in phase 1, and between Dec 12 and Dec 30, 2020, they enrolled 480 participants were enrolled in phase 2. In the combined safety profile of both phases, an adverse reaction occurred in 24%-29% of participants, with most adverse reactions being were mild and moderate in severity - injection site pain (2%-16%). In phase 1, seroconversion of neutralising antibody after the second dose was observed in 100% of participants, and phase 2 in 96.8%-100%. The authors concluded that CoronaVac was well tolerated and safe and induced humoral responses in children and adolescents aged 3-17 years and that the results support the use of 3 · 0 μg dose with a two-immunisation schedule for further studies in children and adolescents [147].

In July 2021, the C4591001 Clinical Trial Group reported on the safety, immunogenicity, and efficacy of the Pfizer/BioNTech’s BNT162b2 Covid-19 vaccine in adolescents. It was a multinational, placebo-controlled, observer-blinded trial. Participants received two injections of 30 μg of BNT162b2 or placebo, 21 days apart, with an objective of noninferiority of the immune response to BNT162b2 in 12-15 year-old participants as compared with that in 16-25 year-old as an immunogenicity objective. Safety and efficacy against confirmed COVID-19 in the 12-15 year-old cohort were assessed. The study was based on 2260 adolescents who confirmed a favourable safety and side-effect profile - injection-site pain in 79%-86%, fatigue in 60%-66%, and headache in 55%-65% and no vaccine-related serious adverse events. After dose 2, the vaccine was not inferior in 12-15 years old participants relative to 16-25 years old participants, with a geometric mean ratio of neutralizing titers of 1.76 (95% confidence interval (CI) = 1.47-2.10). The observed vaccine efficacy was 100% (95% CI = 75%-100%). The study showed that the BNT162b2 vaccine in 12-15 year-old recipients produced a greater immune response than in young adults and was highly effective against COVID-19 [144,153,154]. This study led to the authorization of the Pfizer/BioNTech COVID-19 vaccine for adolescents 12-15 years old in the USA [145,149] and Israel [146].

Towards the end of summer 2021, Moderna’s mRNA-1273 SARS-CoV-2 vaccine was also evaluated in adolescents and the results were published. The authors used phase 2-3 placebo-controlled trial, randomly assigning healthy adolescents aged 12-17 years to receive two injections of the mRNA-1273 vaccine (100 μg in each) or placebo, with two shots given 4 weeks apart. The authors evaluated the safety and the noninferiority of the immune response of mRNA-1273 in adolescents in comparison with young adults (18-25 years of age) in a phase 3 trial. Their secondary objectives were the efficacy of mRNA-1273 in preventing COVID-19 or asymptomatic SARS-CoV-2 infection. The study was based on 3732 participants. The most common adverse reaction was injection-site pain (in 92%-93%), headache (in 45%-70%), and fatigue (in 48%-68%). No serious adverse events were noted, with the geometric mean titer ratio of pseudovirus neutralizing antibody titers in adolescents relative to young adults of 1.08 (95% CI = 0.94-1.24). No cases of COVID-19 were recorded 14 days after the second shot among the vaccinated group [150,151].

Then, a group of Chinese authors reported on the safety and immunogenicity of an inactivated COVID-19 vaccine, BBIBP-CorV, in children and adolescents aged 3-17 years. The design was a randomised, double-blind, controlled, phase 1/2 trial, conducted at Shangqiu City Liangyuan District Center for Disease Control and Prevention in Henan, China. Children were categorized by age groups in 3-5 years, 6-12 years, and 13-17 years. They received three doses of 2 μg, 4 μg, or 8 μg of vaccine or control, 4 weeks apart. Between Aug and Sept 2020, 288 participants were randomly assigned to the vaccine or control group in phase 1, and 720 in phase 2. Adverse reactions were mild to moderate, with pain and fever arising in a minority of participants. BBIBP-CorV also mounted robust humoral responses after two doses, supporting the use of a 4 μg dose and two-shot regimen BBIBP-CorV in phase 3 trials in the population younger than 18 years [155].

Another Chinese study investigated the safety and immunogenicity of a recombinant adenovirus type-5-vectored COVID-19 vaccine with a homologous prime-boost regimen in healthy participants aged 6 years and above. It was a randomised, double-blind, placebo-controlled, phase 2b trial. Participants received the low-dose vaccine, middle-dose vaccine or placebo. Prime-booster regimens were given intramuscularly 56 days apart. A total of 430 participants were enrolled, with 150 participants aged 6-17 years (so-called “MIN cohort”). A single dose in children and adolescents induced higher antibody responses than that elicited by two doses in adults, whereby homologous prime-boost vaccination was safe and tolerable. The authors concluded that the Ad5-vectored COVID-19 vaccine is safe and effective among children and adolescents and that a prime-boost regimen needs further exploration for the Ad5-vectored COVID-19 vaccine [156].

A systematic review of safety, Immunogenicity, and efficacy of COVID-19 vaccines in children and adolescents published in September 2021 found eight published studies with a total of 2852 children and adolescents and 28 ongoing clinical studies, but only two were RCTs, two were case series, and four were case reports. In all studies, the investigated COVID-19 vaccines had good safety profiles in children and adolescents, with injection site pain, fatigue, headache, and chest pain being the most common adverse events [162]. Of the 28 ongoing clinical studies, 23 were interventional studies, conducted in 15 countries. China had 10 ongoing trials, and the USA had 9, with BNT162b2 being the most commonly studied vaccine. The authors concluded that clinical studies of the COVID-19 vaccination in children and adolescents with longer follow-up time, larger sample size, and a greater variety of vaccines were still urgently needed [162].

In November 2021, the “C4591007 Clinical Trial Group” published their study which evaluated the BNT162b2 Covid-19 vaccine in children aged 5 to 11 years. This was a phase 1, dose-finding study and an ongoing phase 2-3 randomized trial. The authors investigated the safety, immunogenicity, and efficacy of two doses of the BNT162b2 vaccine administered 21 days apart in children 6 months to 11 years of age. In phase 2-3 trial, participants were randomly assigned to receive two doses of either the BNT162b2 vaccine or placebo. Immune responses 4 weeks after the second dose were immunologically bridged to those in 16-25 year-olds, and vaccine efficacy against COVID-19 at 7 days or more after the second dose was assessed. A total of 48 children aged 5-11 years received 10 μg, 20 μg, or 30 μg of the BNT162b2 vaccine, 16 children at each dose level. Based on reactogenicity and immunogenicity, a dose level of 10 μg was selected for the phase 2-3 trial. There, a total of 2268 children were randomly assigned to receive the BNT162b2 vaccine or placebo. The BNT162b2 vaccine had a favourable safety profile, with no vaccine-related serious adverse events noted. Four weeks after the second dose, the geometric mean ratio of severe acute respiratory syndrome coronavirus 2 (SARS-CoV-2) neutralizing titers in 5-11 year-olds to those in 16-25 year-olds was 1.04, meeting the prespecified immunogenicity success criterion which had the lower bound of two-sided 95% CI, >0.67, and geometric mean ratio point estimate ≥0.8. Vaccine efficacy was 90.7% (95% CI = 67.7%-98.3%). The authors concluded that COVID-19 vaccination consisting of two 10-μg doses of BNT162b2 administered 21 days apart was safe, immunogenic, and efficacious in children aged 5-11 years [164].

The first large mRNA vaccine trials in children and adolescents have shown that their results were excellent - Pfizer reported 100% vaccine efficacy in children aged 12 to 15 [139] and 90.7% in children aged 5 to 11 years [164]. These results influenced policy and decisions in many countries. At its October 2020 meeting, the Advisory Committee on Immunization Practices (ACIP) approved the 2021 Recommended Child and Adolescent Immunization Schedule for Ages 18 Years or Younger; weeks later, after Emergency Use Authorization of Pfizer-BioNTech COVID-19 vaccine by the Food and Drug Administration (FDA), ACIP issued an interim recommendation for use of Pfizer-BioNTech COVID-19 vaccine in persons aged ≥16 years at its December 12, 2020, meeting. In addition, ACIP approved an amendment to include COVID-19 vaccine recommendations in the child and adolescent immunization schedule. After the Emergency Use Authorization (EUA) of Moderna COVID-19 vaccine by the FDA, ACIP issued an interim recommendation for use of Moderna COVID-19 vaccine in persons aged ≥18 years at its December 19, 2020, emergency meeting [140,142]. On May 10, 2021, FDA expanded the EUA for the Pfizer-BioNTech COVID-19 vaccine to include adolescents aged 12-15 years. On May 12, 2021, ACIP issued an interim recommendation for use of the Pfizer-BioNTech COVID-19 vaccine in adolescents aged 12-15 years for the prevention of COVID-19 [143].

## VACCINE UPTAKE IN MINORS AND POPULATION-LEVEL EFFECTIVENESS

By May 12, 2021, approximately 141.6 million doses of the Pfizer-BioNTech COVID-19 vaccine had been administered to persons aged ≥16 years in the USA. As of July 30, 2021, among the three COVID-19 vaccines authorized for use in the United States, only the Pfizer-BioNTech BNT162b2 mRNA COVID-19 vaccine was authorized for adolescents aged 12-17 years. Beginning in June 2021, cases of myocarditis and myopericarditis (hereafter, myocarditis) after Pfizer-BioNTech vaccination were reported, primarily among young males after receipt of the second dose. On June 23, 2021, CDC's ACIP reviewed available data and concluded that the benefits of COVID-19 vaccination to individual persons and the population outweigh the risks for myocarditis. As a result, ACIP recommended continued use of the vaccine in persons aged ≥12 years. To further characterize the safety of the vaccine, adverse events after receipt of the Pfizer-BioNTech vaccine were reported to the Vaccine Adverse Event Reporting System (VAERS). Furthermore, adverse events and health impact assessments that were reported in v-safe (a smartphone-based safety surveillance system) were reviewed for US adolescents aged 12-17 years during December 14, 2020-July 16, 2021. As of July 16, 2021, approximately 8.9 million US adolescents aged 12-17 years had received the Pfizer-BioNTech vaccine. CDC and FDA continued to monitor vaccine safety and provide data to ACIP to guide COVID-19 vaccine recommendations [148].

On August 23, 2021, FDA approved a Biologics License Application for use of the Pfizer-BioNTech COVID-19 vaccine, Comirnaty (Pfizer, Inc.), in persons aged ≥16 years. The ACIP COVID-19 Vaccines Work Group's conclusions regarding the evidence for the Pfizer-BioNTech COVID-19 vaccine were presented to ACIP at a public meeting on August 30, 2021. The additional information increased certainty that benefits from prevention of asymptomatic infection, COVID-19, and associated hospitalization and death outweighs vaccine-associated risks. On August 30, 2021, ACIP issued a recommendation for use of the Pfizer-BioNTech COVID-19 vaccine in persons aged ≥16 years for the prevention of COVID-19 [157].

As of November 2, 2021, approximately 248 million doses of the Pfizer-BioNTech COVID-19 vaccine had been administered to persons aged ≥12 years in the United States. On October 29, 2021, FDA issued a EUA amendment for a new formulation of the Pfizer-BioNTech COVID-19 vaccine for use in children aged 5-11 years, administered as 2 doses (10 μg, 0.2 mL each), 3 weeks apart. On November 2, 2021, the Advisory Committee on Immunization Practices (ACIP) issued an interim recommendation for use of the Pfizer-BioNTech COVID-19 vaccine in children aged 5-11 years for the prevention of COVID-19. The Pfizer-BioNTech COVID-19 vaccine showed high efficacy (>90%) against COVID-19 in children aged 5-11 years [166].

Both Pfizer-BioNTech and Moderna vaccines are mRNA vaccines encoding the stabilized prefusion spike glycoprotein of the SARS-CoV-2 virus. Both mRNA vaccines were authorized and recommended as a 2-dose schedule, with second doses administered 21 days (Pfizer-BioNTech) or 28 days (Moderna) after the first dose. Besides those two vaccines, only a handful of others have been tested in young people over the age of 12, including two Chinese vaccines made by Sinovac and Sinopharm. Other studies are expected to report results in young people over the age of 12 soon, including the Zydus Cadila vaccine and the Covaxin inactivated coronavirus vaccine, both made in India. Thus far, the vaccines seem to be safe in adolescents, while companies have moved on to carrying out clinical trials in children as young as six months old [57,166].

Given that nearly one-third of adolescents aged 12-17 years who needed to be hospitalized with COVID-19 between March 2020 and April 2021 in the USA required intensive care, and about 5% even required endotracheal intubation and mechanical ventilation, by July 31, 2021, 42.4% of US adolescents aged 12-17 years received at least one dose of COVID-19 vaccine and 31.9% received both doses. The first dose coverage ranged from 20.2% in Mississippi to 70.1% in Vermont, and the coverage was the highest in the age group 16-17 years (50.6%), followed by 14-15 years (40.9%), and 12-13 (36.0%) [152].

In Israel, the BNT162b2 vaccine against severe acute respiratory syndrome coronavirus 2 was approved for use in adolescents in June 2021, shortly before an outbreak of the Delta variant. Short-term vaccine effectiveness after the first dose was about 55% and after the second dose about 90% [158,160]. In the US, a randomized placebo-controlled trial demonstrated an efficacy of Pfizer-BioNTech vaccine of nearly 100% (95% CI = 75.3%-100%) in preventing outpatient COVID-19 in children aged 12-15 years. In a test-negative, case-control study at 19 pediatric hospitals in 16 states during June 1-September 30, 2021, the effectiveness of 2 doses against COVID-19 hospitalization was assessed among 12-18-year-olds. A total of 464 hospitalized persons were included in the study, 179 case-patients and 285 controls, with a median age of 15 years. Among them, 72% had at least one underlying condition, including obesity, and 68% attended in-person school. Similarly to the observations in Israel, effectiveness of 2 doses was 93% (95% CI = 83%-97%) during the Delta period [161,168].

The next set of data that will be awaited is the information on vaccine roll-out among the age group 5-11 years. The administration of the vaccine in this age group began in early November 2021 [163,165], and the expectation is that the Pfizer-BioNTech vaccine will provide 90% protection against infection in this age group of children [167].

In Israel, the effectiveness of BNT162b2 Vaccine among Adolescents aged 12-18 years was studied among 184 905 vaccinated adolescents, where 94 354 vaccine recipients were matched with 94 354 unvaccinated controls and the frequency of polymerase-chain-reaction (PCR) testing for SARS-CoV-2 was similar in the vaccinated and unvaccinated populations. The estimated vaccine effectiveness against documented SARS-CoV-2 infection was 59% (95% CI = 52%-65%) on days 14-20 after the first dose, 66% (95% CI = 59%-72%) on days 21-27 after the first dose, and 90% (95% CI = 88%-92%) on days 7-21 after the second dose. The estimated vaccine effectiveness against symptomatic COVID-19 was 57% (95% CI = 39%-71%) on days 14-20 after the first dose, 82% (95% CI = 73%-91%) on days 21-27 after the first dose, and 93% (95% CI = 88%-97%) on days 7-21 after the second dose. This study showed that the BNT162b2 mRNA vaccine was highly effective in the first few weeks after vaccination against both documented infection and symptomatic COVID-19 with the delta variant among adolescents between the ages of 12-18 years [169].

## POSSIBLE SIDE EFFECTS OF COVID-19 VACCINES IN CHILDREN AND YOUNG PEOPLE

The literature on the possible side effects of COVID-19 vaccines in children and young people is still sparse [170-190]. First reports appeared in the second half of 2021. A case series on adolescents receiving the BNT162b2 mRNA COVID-19 vaccine from 3 pediatric medical centres in Israel [170] described 7 males who were 16-18 years old, of Jewish descent, with chest pain that began 1-3 days following vaccination. In six of the seven patients, symptoms began after the second dose, and in one patient they followed the first dose. All cases were mild and didn’t require specific support, either cardiovascular or respiratory. A comparison with previous years showed that the incidence of perimyocarditis during the vaccination period was elevated. Seven further cases of acute myocarditis or myopericarditis in healthy male adolescents from the USA showed a very similar picture: chest pain within 4 days after the second dose of Pfizer-BioNTech COVID-19 vaccination in all cases, where five also had a fever. COVID-19 was ruled out by PCR and MIS-C by detailed diagnosis. Six also had negative SARS-CoV-2 antibody assays, suggesting no previous infection with SARS-CoV-2, and all seven had an elevated troponin. On cardiac MRI, they presented late gadolinium enhancement, characteristic of myocarditis. All seven patients rapidly recovered. In three, nonsteroidal antiinflammatory drugs were used, while four received intravenous immunoglobulin and corticosteroids [171].

Further reports supported this initial observation, with remarkably similar features. Five male patients in Israel with a median age of 23 had post- BNT162b2 vaccine myocarditis [172], and eight further adolescents in the USA presented with perimyocarditis within 4 days of receiving a dose of BNT162b2 vaccine [175]. A series of 25 children, aged 12-18 years, were diagnosed with probable myopericarditis after COVID-19 mRNA vaccination at eight US centres between May and June 2021. Again, in 88%, the cases followed the second dose of vaccine, and chest pain was the most common presenting symptom in them all - after a median of 2 days (range from <1 to 20 days) following vaccination. All affected children had an elevated plasma troponin concentration, 92% showed normal cardiac function, and 94% had late gadolinium enhancement. Most were treated with ibuprofen or an equivalent nonsteroidal anti-inflammatory drug and everyone was well within a week [177].

Another case series of 15 minors from a single institution in the USA [179] showed that 14 were males with a median age of 15 years (range: 12-18 years). Their symptoms started 1-6 days after vaccination and included chest pain in all patients, fever in 67%, myalgia in 53%, and headache in 40%, with troponin levels elevated in all patients at admission. Decreased left ventricular ejection fraction was present in 20% and abnormal global strain in 33%. Late gadolinium enhancement was seen in 80% of patients. No patient required ICU and the median hospital length of stay was two days (range 1-5). Only one patient had persistent borderline low LV systolic function on echocardiogram, with an ejection fraction of 54%, after 14 days of follow-up [179]. Then, two further cases of a similar presentation were reported [185], but larger case series was needed.

In a retrospective multicenter study across 16 US hospitals, with patients <21 years of age, the acronym C-VAM was introduced for “coronavirus disease 2019 vaccination-associated myocarditis” and a series of 63 patients with a mean age of 15.6 years was presented [180]. Again, 92% were male and all of them but one presented C-VAM after the second dose. Only four patients had significant dysrhythmia; 14% had mild left ventricular dysfunction on echocardiography, and 88% met the diagnostic CMR Lake Louise criteria for myocarditis. Late gadolinium enhancement on CMR was more prevalent in comparison with MIS-C. No one required ICU support and there were no deaths. After about a month of follow-up, the symptoms, arrhythmias, and ventricular dysfunction resolved. Still, a long-term follow-up will be needed to document any persisting consequences. Mechanisms of these myocardial tissue changes should also be understood [180].

By May 31, 2021, early reports of myocarditis prompted the Israeli Ministry of Health to initiate active surveillance. Using the Brighton Collaboration definition, they analyzed the occurrence of myocarditis by computing the risk difference for the comparison of the incidence after the first and second vaccine doses, which were given 21 days apart, and by calculating the standardized incidence ratio of the observed-to-expected incidence. They found 304 persons with symptoms of myocarditis, but 21 were reclassified; of the remaining 283 cases, 142 occurred after the BNT162b2 vaccination, and 136 diagnoses were either definitive or probable. In 95% of cases, the clinical presentation was mild, but one fulminant case was fatal. The overall risk difference between the first and the second dose was 1.76 per 100 000 persons, with the largest risk among males aged 16-19 years (13.73 per 100 000 persons). As compared to historical incidence data, the standardized incidence ratio was 5.34. It was the highest after the second dose in males aged 16-19 years (13.60). When fully vaccinated recipients were compared to unvaccinated, their increased risk was 2.35, but the rate ratio was the highest in males aged 16-19 years whose absolute risk was about 1 in 6500 [186].

After reports of myocarditis and pericarditis in mRNA vaccine recipients, which predominantly occurred in young males after the second dose, a US Advisory Committee on Immunization Practices (ACIP) meeting was rapidly convened to review reported cases of myocarditis and pericarditis and discuss the benefits and risks of mRNA COVID-19 vaccination in the United States. On June 23, 2021, after reviewing available evidence including that for risks of myocarditis, ACIP determined that the benefits of using mRNA COVID-19 vaccines under the FDA's Emergency Use Authorizations (EUA) outweigh the risks in adolescents and young adults. The EUA has been modified to include information on myocarditis after receipt of mRNA COVID-19 vaccines, with the fact sheets to be provided before vaccination; in addition, CDC has developed patient and provider education materials about the possibility of myocarditis and symptoms of concern, to ensure prompt recognition and management of myocarditis [173,176]. Importantly, none of the patients fulfilled the criteria for multi-system inflammatory syndrome or Kawasaki-like disease and there was no evidence of acute SARS-CoV-2 infection [184].

By mid-July 2021, the CDC’s Vaccine Adverse Event Reporting System (VAERS) in the US collected 9246 reports of postvaccination adverse events among the adolescents aged 12-17 years or about 1 per 1000 vaccinated. More than 90% of those reports were not for serious symptoms and they involved dizziness, fainting, nausea, headache, and fever. A total of 863 events were considered serious, including chest pain, increased troponin levels, and myocarditis. Myocarditis disproportionately affected males and it was reported in 397 cases, representing 4.3% of all VAERS reports. Fourteen deaths were also reported, but there was no apparent pattern that would indicate that vaccination was the cause, although the cause of death for 6 adolescents was “unknown or pending”. About half of the recipients of the Pfizer-BioNTech‘s mRNA vaccine, the only one licensed for this age group at the time, reported systemic reactions after the first dose, and about two-thirds after the second dose. They usually occurred during the first day after vaccination. The reported symptoms included injection site pain, fatigue, headache, and muscle pain. Fever was present in about one-third of the week after the second dose, while about one-quarter were unable to perform daily activities the day after their second shot [183].

Another country that was quick to report the side effects of the COVID-19 Pfizer-BioNTech mRNA vaccine in children aged 12-18 years was Saudi Arabia. An internet-based study using a self-administered online survey was prone to self-selection bias and it reported at least one side effect in 60% of participants. Among them, 90% reported pain or redness at the site of injection (90%), 67% fatigue, 59% fever, 55% headache, 21% nausea or vomiting, and 20% chest pain and shortness of breath, with only 2% reporting joint or bone pain [189]. Meanwhile in Scandinavia, Sweden, Norway, and Finland suspended the use of the Moderna vaccine in young people “as a precaution” on October 7, 2021, after reports of possible rare side effects. This applied to anyone born in 1991 or later. In Norway, the use of Moderna’s vaccine was suspended in those under 18 years of age, who were offered the Pfizer vaccine instead. The cause of this decision were the reports of an increased risk of myocarditis and pericarditis, which seemed to occur more often after the Moderna vaccine than Pfizer’s [187].

In addition to myocarditis, other very rare side effects of vaccination underwent scrutiny. Cerebral venous thrombosis has been studied in adults through a multicentre cohort study in the UK, but its surveillance should be extended to minors, too. In the adults, the new syndrome was termed “vaccine-induced immune thrombotic thrombocytopenia” (VITT), with cerebral venous thrombosis being the most common manifestation. Between April 1 and May 20, 2021, the authors received data on 99 patients from collaborators in 43 hospitals across the UK, with 70 eventually having the VITT confirmed. The primary outcome, which was defined as “death or dependency”, occurred more frequently in patients with VITT-associated cerebral venous thrombosis - in 33 of 70 patients. Non-heparin anticoagulants and intravenous immunoglobulin showed some effectiveness against these outcomes [178]. Opinions were expressed that children can also have thrombosis with COVID-19 and that this potential problem should be closely monitored [174].

In Japan, a case of new-onset pediatric nephrotic syndrome following Pfizer-BioNTech SARS-CoV-2 vaccination was described. Several adult cases of minimal change nephrotic syndrome have already been reported earlier after the vaccine, but no warnings were published about children. A 15-year-old boy had no underlying diseases and he developed eyelid oedema four days after vaccination, with peripheral oedema of the lower extremities four days later. Treatment involved 60 mg of oral daily prednisolone, and complete remission was achieved within 12 days with no residual complications (eg, hypertension or acute kidney injury) [188].

It has also been proposed in adults that MIS may occur after SARS-CoV-2 vaccination, which is then no longer branded as “MIS-C”, but rather as “MIS-V”. A multisystem inflammatory syndrome was reported in an 18-year-old adolescent after the SARS-CoV-2 vaccine from Pfizer/BioNTech (BNT162b2). The key clinical features were fever for 3 consecutive days, pericardial effusion, elevated CRP/NT-BNP/Troponin T/D-dimers, cardiac involvement, and positive SARS-CoV-2 antibodies. The disease started 10 weeks after the second dose of the vaccine. A pericardial effusion was diagnosed by echocardiography, with the CRP, NT-BNP, D-dimers and troponin T levels all elevated [190].

In the initial study of the safety and tolerability of the BNT162b2 vaccine in extremely vulnerable children aged 12-15 years, eight events were reported in six children after the first dose, and they all resolved within less than 3 days. Those were mild rash, headache, diarrhoea, presumed sore throat, neck pain, difficulty sleeping and decreased levels of blood glucoses. After the second dose, eight additional events occurred among five children: diarrhoea, vomiting, armpit swelling, and blisters around the mouth, which were not thought to be related to vaccination [182]. In another study, that looked into the safety of the administration of Pfizer-BioNTech COVID-19 vaccine in youth and young adults with a history of acute lymphoblastic leukaemia (ALL) and allergy to polyethene glycol (PEG)-asparaginase, the fact that the mRNA vaccines contain PEG as a stabilizer was specifically considered. Most patients treated with contemporary combination treatments for ALL receive PEG-asparaginase (PEG-ASNase), but 10%-30% develop allergic reactions. The authors concluded that optimizing access and safety for vaccine administration for these patients is critical and described a process developed to support vaccination among young people with a history of PEG-ASNase allergy [181].

## CONCLUSIONS

Research to characterise SARS-CoV-2 infection and COVID-19 in children and young people rapidly evolved during 2020 and 2021 and led to recommendations from several professional societies and authorising bodies that vaccinating children and young people should be safe and effectively protect them from COVID-19. Ethical and legal aspects of vaccinating minors were debated, leading to a vivid discussion in the scientific literature over the arguments whether children and young people should also be included in COVID-19 vaccine trials. The emergence of new variants of SARS-CoV-2 which had increased virulence added another argument to the debate as it became clear that very high population-based vaccination coverage would be required to control the pandemic. A body of literature then explored caregivers’ willingness to vaccinate minors and the extent of vaccine hesitancy that could meet national programmes of vaccinating children and young people. Eventually, vaccines were approved for those aged 16-18 years; then, the first vaccine safety and efficacy trials were conducted in the adolescents aged 12-15 years; and finally, children under the age of 12 were also included. Population-level effectiveness of this vaccination was then determined, and possible side effects such as myocarditis were documented and discussed. However, the debate on the benefits will continue given rapidly waning immunity in children and young people, the appearance of the new variants of the SARS-CoV-2, and the proposals in some countries of making COVID-19 vaccines mandatory among minors.
